# An agmatine-inducible system for the expression of recombinant proteins in *Enterococcus faecalis*

**DOI:** 10.1186/s12934-014-0169-1

**Published:** 2014-12-04

**Authors:** Daniel M Linares, Marta Perez, Victor Ladero, Beatriz del Rio, Begoña Redruello, Mª Cruz Martin, María Fernandez, Miguel A Alvarez

**Affiliations:** Instituto de Productos Lácteos de Asturias, IPLA-CSIC, Paseo Río Linares s/n, 33300 Villaviciosa, Asturias Spain

**Keywords:** *Enterococcus*, Overexpression, Expression vector, Agmatine

## Abstract

**Background:**

Scientific interest in *Enterococcus faecalis* has increased greatly over recent decades. Some strains are involved in food fermentation and offer health benefits, whereas others are vancomycin-resistant and cause infections that are difficult to treat. The limited availability of vectors able to express cloned genes efficiently in *E. faecalis* has hindered biotechnological studies on the bacterium’s regulatory and pathogenicity-related genes. The agmatine deiminase (AGDI) pathway of *E. faecalis*, involved in the conversion of agmatine into putrescine, is driven by a response inducer gene *aguR*.

**Results:**

This study describes that the exposure to the induction factor (agmatine) results in the transcription of genes under the control of the *aguB* promoter, including the *aguBDAC* operon. A novel *E. faecalis* expression vector, named pAGEnt, combining the *aguR* inducer gene and the *aguB* promoter followed by a cloning site and a stop codon was constructed. pAGEnt was designed for the overexpression and purification of a protein fused to a 10-amino-acid His-tag at the C-terminus. The use of GFP as a reporter of gene expression in *E. faecalis* revealed that under induction with 60 mM agmatine, fluorescence reached 40 arbitrary units compared to 0 in uninduced cells.

**Conclusion:**

pAGEnt vector can be used for the overexpression of recombinant proteins under the induction of agmatine in *E. faecalis,* with a close correlation between agmatine concentration and fluorescence when GFP was used as reporter.

## Background

Members of the genus *Enterococcus* are found throughout the normal gut of vertebrates and insects and are commonly associated with fermented foods. In artisanal Mediterranean cheeses they are believed important in the development of flavour and aroma [1-3]. *Enterococcus* has been the subject of much research given its members’ potential as biopreservatives. Some strains isolated from cheese produce enterocins (bacteriocins); their bactericidal activity may be effective against food-borne pathogens such as *Listeria monocytogenes*, *Vibrio cholerae*, *Staphylococcus aureus, Clostridium botulinum, Salmonella enterica* and *Bacillus cereus*, as well as against spoilage microorganisms [4-8]. Indeed, non-pathogenic enterococci have promising commercial potential [9]. They are already used as starter cultures in certain artisanal food fermentations and are commercially available as probiotics [10-12]. For example, *Enterococcus faecalis* Symbioflor 1 clone DSM 16431 is included in a commercial probiotic product with potential benefits for human health - it has been used for over 50 years with no reports of infection [13]. Given their ability to improve the microbial equilibrium of the intestine, some strains of *Enterococcus faecium* have also been used as probiotic adjunct cultures in the making of Cheddar cheese [7]. In addition, enterococci have been used as animal feed additives in the European Union [14], and as probiotics for farm animals destined for human consumption [15-17]. The well-studied probiotic strain *E. faecium* SF68*,* produced in Switzerland under the name Cylactin® (DSM Nutritional Products Ltd.), is currently authorised by the European Food Safety Authority (EFSA) for use in goat and kid feed for the treatment of diarrhoea [18,19].

Enterococci can, however, also be harmful. Some strains have been studied because of their production of biogenic amines (mostly tyramine and putrescine) in fermented foods [20-23]. Other strains have virulence factors and are recognised as emerging pathogens responsible for serious clinical infections [24,25]. Enterococci are also among the most important multidrug-resistant organisms affecting immunocompromised patients [26]. This bacterial group can acquire genetic determinants that confer resistance to antibiotics and it is particularly concerning the acquisition of vancomycin resistance [27]. Indeed, the emergence of vancomycin-resistant enterococci (VRE) has alarmed the global infectious diseases community given the few options left for the management of their associated diseases. The transfer of resistance genes from enterococci to other strains, the presence of selection pressures for VRE proliferation, and the rapid expansion of resistant populations are matters of great concern. The prevalence of VRE has been reported for Europe, Asia, Australia, South America and some African countries [26].

Our knowledge of the regulation of virulence factors in *E. faecalis*, and of the environmental signals that contribute towards pathogenicity, remains poor [28]; a pressing need exists to understand this infectious agent at the genetic and physiological level. Studies in these areas, however, require molecular and genetic tools be available. For many investigations, e.g., on genetic regulation, knock-out complementation, the heterologous expression of bacteriocins in bacteriocin-negative hosts, or research on membrane permeases, the overexpression of genes is often required [29-31]. For *Enterococcus*, some expression vectors based on bacteriocin-inducible promoters have been developed, but these require the genes encoding the kinase and regulator proteins supplied *in trans* [32]. Beside this, the widely used *Lactococcus lactis* NICE system is suitable for use in other Gram-positive species including *Enterococcus*, either by using two simultaneous compatible plasmids - one harbouring the expression cassette and the other carrying the *nisRK* genes- [33], or as a single-plasmid expression vector including both cassettes [34]. In addition, other *E. faecalis* expression systems based on rhamnose as inducer [35] or under control of the pheromone cCF10 [36] have been described. Yet, the molecular genetic tools available for *E. faecalis* are more limited than for some other microorganisms. In recent years, research has therefore focused on the development of alternative Gram-positive bacterial expression systems for the production of industrially important proteins [36]. The development of controlled gene expression systems for homologous and heterologous gene expression therefore remains an important goal.

**Figure 1:**
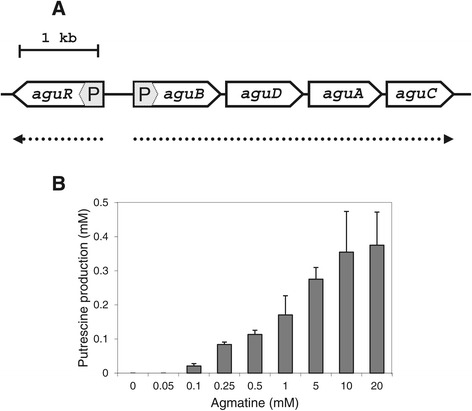
**Genetic organization of the** ***AGDI*** **cluster of** ***E. faecalis*** **V583 and putrescine production. A**. The AGDI cluster of *E. faecalis* is composed of five genes, *aguR* encoding a putative regulator, followed by *aguB*, *aguD*, *aguA* and *aguC* which encode the putrescine biosynthesis pathway (accession number NC_004668). The P_*aguR*_ and P_*aguB*_ promoters are shaded and their predicted transcripts are indicated below (dotted arrows). **B**. Putrescine production by *E. faecalis* V583 in 24 h cultures grown with 0, 0.05, 0.1, 0.25, 0.5, 1, 5, 10 or 20 mM agmatine. Values are the means of three independent assays and error bars denote standard deviation.

The agmatine deiminase (AGDI) genes cluster of *E. faecalis* contains the genes necessary for the biosynthesis of putrescine from agmatine. *aguD* encodes for the agmatine/putrescine antiporter, *aguA* encodes for the agmatine deiminase, *aguB* encodes for the putrescine transcarbamylase, and *aguC* encodes for a specific carbamate kinase [22]. Overall, the locus is organized in an operon constituted by the metabolic genes *aguB*, *aguD*, *aguA* and *aguC*, which are cotranscribed in a single mRNA from the *aguB* promoter (P_*aguB*_), in a divergent orientation with the putative regulator gene (*aguR*) that has its own promoter (P_*aguR*_) [37] (Figure 1A). Our working hypothesis is that AguR would activate the transcription from P_*aguB*_ and therefore, we could construct a genetic expression system inducible by the addition of agmatine in the culture medium.

In the present work, by combining the *aguR* inducer gene and the P_*aguB*_ promoter (with its natural ribosome binding site), a new agmatine-inducible expression system – the “agmatine controlled expression (ACE)” system - was developed. This system was successfully used in the production of green fluorescent protein (GFP) in *E. faecalis* V583, and offers a practical and straightforward method for the heterologous expression of other recombinant proteins in *E. faecalis*.

## Results

### Influence of agmatine on putrescine production

Since agmatine is the substrate for putrescine biosynthesis in the AGDI pathway, the potential of *E. faecalis* V583 to produce putrescine in GM17 supplemented with a range of different concentrations of agmatine (0, 0.05, 0.1, 0.25, 0.5, 1, 5, 10 or 20 mM) was investigated. Liquid cultures were inoculated (2% v/v) with overnight cultures and incubated at 37°C for 24 h. Accumulation of putrescine in the supernatants was analysed by UPLC™ (Figure 1B). As expected, a positive correlation was seen between agmatine supplementation and putrescine accumulation in the extracellular medium. Putrescine was first detectable in the supernatant after treatment with 0.1 mM agmatine. Above this concentration, putrescine accumulation was gradually increased with increasing agmatine concentration.

### Transcriptional regulation of the AGDI operon by agmatine

The expression profile of the genes of the AGDI operon was analysed by RT-qPCR in GM17 media with supplementation of 0, 0.05, 0.1, 0.25, 0.5, 1, 5, 10 or 20 mM agmatine. RNA samples were taken at the end of the exponential phase of growth (t = 6 h; i.e., once putrescine production had started) and mRNA levels compared. Since *aguB*, *aguD*, *aguA* and *aguC* are coexpressed in a single transcript, the expression of *aguA* is shown as representative of all *aguBDAC* mRNA.

**Figure 2:**
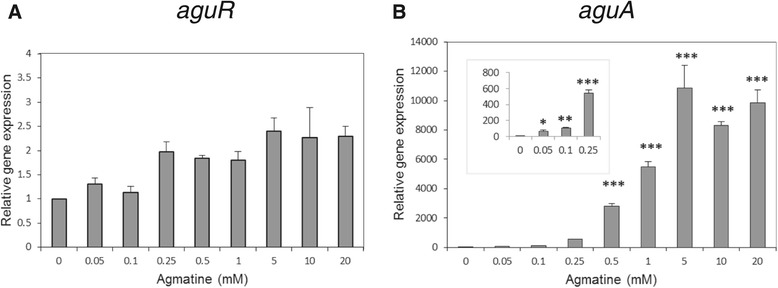
**Relative gene expression of** ***aguR*** **(A) and** ***aguA*** **(B) in cultures grown with 0, 0.05, 0.1, 0.25, 0.5, 1, 5, 10 or 20 mM agmatine.** The expression of each gene at 0 mM was independently normalised to 1, and used as the reference condition. Values shown are the means of three independent assays and error bars denote standard deviation. Asterisks indicate statistically significant differences in relative expression levels with respect to the no-agmatine condition (*P < 0.05; **P < 0.01; ***P < 0.005; Student *t* test).

The expression level of *aguR* remained almost constant under all conditions of agmatine supplementation (Figure 2). However, the expression of *aguBDAC* was strictly dependent on agmatine, and a significant increase in *aguBDAC* mRNA was observed under agmatine supplementation. In fact, agmatine induction of *aguR* transcription (approximately 2-fold increase in relative transcript levels) was much lower than that of the *aguBDAC* operon (approximately 10,000-fold increase in relative transcript levels) (Figure 2).

### Construction of the expression vector pAGEnt

**Figure 3:**
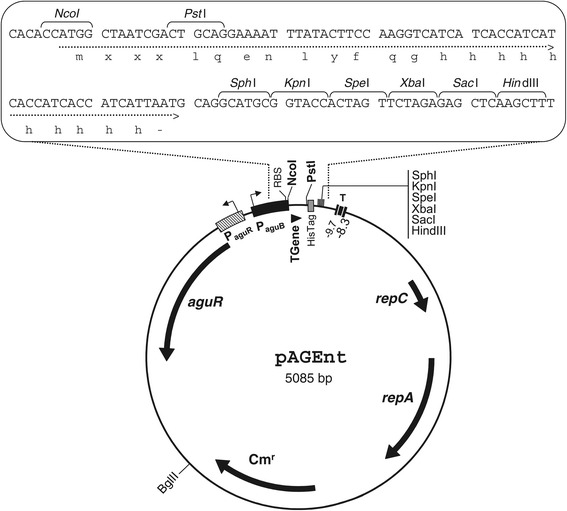
**Genetic map of the expression vector pAGEnt and zoom-in of the expression cassette.** *repC* and *repA*, replication genes; cm^r^, chloramphenicol resistance gene; *aguR*, gene encoding the regulatory protein AguR; P_*aguR*_, *aguR* promoter; RBS, ribosome binding site; T, transcription terminator; P_*aguB*_, *aguB* agmatine-inducible promoter; TGene, open reading frame for target gene of interest; His-tag; C-terminal histidine tag. Representative restriction sites are indicated.

The core of the lactococcal vector pNZ8048 [38,39], which includes the replication cassette and the chloramphenicol resistance marker, was used as a starting point for the construction of the pAGEnt vector. First, a fragment of the chromosomal AGDI cluster from *E. faecalis* V583 including the *aguR* promoter (P_*aguR*_), the *aguR* gene, and the *aguB* promoter (P_*aguB*_), was amplified by PCR using the primers indicated in Table 1, and cloned into the *Bgl*II-*Nco*I sites of pNZ8048. A fragment including the multicloning site and the histidine tag (His-tag) sequence was then PCR-amplified using plasmid pNZErmC [40] as a template, employing the primers Expvfor1 and Expvrev1. The products were digested and cloned into the *Nco*I-*Hind*III sites of the previous vector, thus providing plasmid pAGEnt (Figure 3).

**Table 1:** **Oligonucleotides used**

**Primer**	**Function**	**Sequence (5′ to 3′)**
*aguAq F*	*aguA* expression analysis (F)	TTGTGCCGCTTCATAAAATGG
*aguAq R*	*aguA* expression analysis (R)	CACCTGGTGAAGTGGCTTGTATT
*aguRq F*	*aguR* expression analysis (F)	CGGGTTCATCTGATTGATTTTCTTC
*aguRq R*	*aguR* expression analysis (R)	CGTGATTTTCCTCTGTCGGTTCTT
*recA F*	*recA* internal control (F)	CAAGGCTTAGAGATTGCCGATG
*recA R*	*recA* internal control (R)	ACGAGGAACTAACGCAGCAAC
*EFV583-tufF*	*tuf* internal control (F)	CAGGACATGCGGACTACGTTAA
*EFV583-tufR*	*tuf* internal control (R)	TAGGACCATCAGCAGCAGAAAC
*AguR-EntBglII*	Cloning P_*aguR*_-AguR-P_*aguB*_ cassette (F)	CCCCAGATCT TTAAAAAGAAACAAGGTGGTGGCCG
*AguR EntNco*	Cloning P_*aguR*_-AguR-P_*aguB*_ cassette (R)	CCCCATGG TGTGTTCCTCCTAAAAGTTGTTTTTG
*Expvfor1*	Insertion of His-tag (F)	CACACACACCCATGGCTAATCGACTGCAGGAAAATTTATACTTCCAAGGTC
*Expvrev1*	Insertion of His-tag (R)	CTATCAATCAAAGCAACACGTG
*GfF1*	Cloning of GFP (F)	CACACACACCCATGGAATTCAGTAAGGGAGAAGAACTTTTC
*GfR1*	Cloning of GFP (R)	CACACACACCTGCAG ACTAGTTTTGTAGAGCTCATCCATGC

F, forward; R, reverse; P_*aguR*_ , *aguR* promoter; P_*aguB*_ , *aguB* promoter; *recA*, recombinase A; *tuf*, elongation factor Tu.

The resulting pAGEnt vector offers the possibility of traditional cloning without the His-tag, i.e., the insertion of the gene of interest into the extensive range of sites provided by the multiple cloning site, or the fusion of an optional C-terminal 10x His-Tag by cloning the target gene specifically into the *Nco*I-*Pst*I sites. The latter alternative enables the insertion of a promoterless gene into the correct frame, fused to the His-tag and ready for expression. Subsequent protein purification is therefore possible. Thus, this vector can be used for gene overexpression, protein immunodetection, and purification purposes.

### Controlled heterologous expression of *gfp*

To test the usefulness of pAGEnt in the expression of a foreign gene, tests were performed to determine whether the *aguR*/P_*aguB*_ cassette was able to drive the expression of heterologous genes. The promoterless gene *gfp* encoding green fluorescent protein was cloned (using the primers indicated in Table 1) as a reporter into the *Nco*I-*Pst*I sites of pAGEnt, generating the vector pAGEnt-GFP. Fluorescence measurements revealed that, upon the addition of the induction factor agmatine (20 mM), AguR significantly enhanced the expression of *gfp* by up to 10.13 arbitrary units. Parallel cultures harbouring the empty vector pAGEnt, or the vector pAGEnt-GFP with no agmatine present, provided negative controls; expression of the gene of interest was undetectable (<0.5 arbitrary units).

### Sensitivity of the pAGEnt system to the inducer: dose–response curve

**Figure 4:**
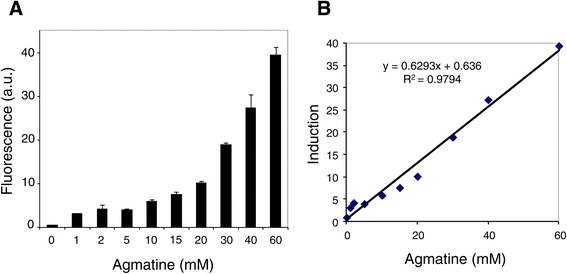
**Sensitivity of the** ***aguR*** **/P**_***aguB***_ **expression system to the inducer. A**. Induction strength of *gfp* in *E. faecalis* V583 cultures containing pAGEnt-GFP under induction with a range of agmatine concentrations. Cultures harbouring the empty vector pAGEnt, or the vector pAGEnt-GFP with no agmatine present, were carried in parallel as negative controls. Error bars: mean standard deviation of three independent experiments. **B**. Correlation between inducer concentration (mM) and reporter fluorescence (arbitrary units).

The control afforded by the agmatine-induced expression system in the production of recombinant proteins was tested. The production of GFP in cultures, induced with a range of agmatine concentrations (between 0 and 60 mM), was analysed by whole-cell fluorescence. Figure 4 shows an increase in fluorescence with even low concentrations of agmatine (1 mM). Induction levels increased with the agmatine concentration; indeed, a close correlation was seen between agmatine concentration and fluorescence (R^2^ = 0.979). In the presence of 60 mM agmatine, fluorescence reached 40 arbitrary units compared to 0 in uninduced cells. The virtual lack of fluorescence with 0 mM agmatine demonstrates the absence of any leaky activity of the promoter P_*aguB*_.

### Toxicological assessment of agmatine in *Enterococcus faecalis*

Agmatine has been reported to act as an anti-proliferative agent in several non-intestinal mammalian cell models [41]. Since high induction concentrations were tested in the present work, assays were performed to see whether these affected bacterial viability.

**Figure 5:**
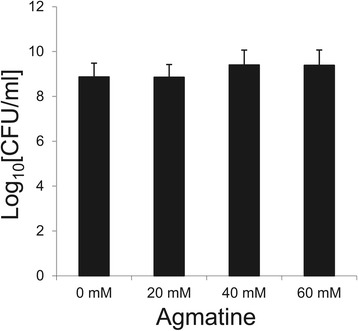
**Susceptibility of** ***E. faecalis*** **V583 to agmatine.** *E. faecalis* cultures were grown in liquid GM17 supplemented with 0 mM, 20 mM, 40 mM or 60 mM agmatine. After 24 h of incubation, serial dilutions of the suspension were prepared and immediately plated to determine the number of viable bacteria (cfu/ml). The number of bacteria observed was within the range of 30 to 300 cfu per plate. Error bars represent standard deviation of three independent experiments.

*E. faecalis* cultures were grown in liquid GM17 supplemented with, 20, 40 or 60 mM agmatine. After 24 h incubation, serial dilutions of each suspension were prepared and immediately plated to determine the number of viable bacteria (colony forming units per millilitre [cfu/ml]). Under all conditions, the average viable count ranged from 8.85 to 9.39 log_10_[cfu/ml] units; no significant differences were seen between the different agmatine-treated and untreated cultures (Figure 5). Thus, agmatine has no toxic effect on *E. faecalis* V583 cell viability after 24 hours of exposure*,* even at the high concentrations required for maximal induction.

## Discussion

Some strains of *E. faecalis* have commercial uses, e.g., as probiotics that promote a beneficial gut environment [42] and in food fermentation processes [1,43-45]. Other strains, however, can pose food safety problems and yet others are a leading cause of opportunistic, hospital-acquired infections (including urinary tract infections, septicaemia, bacteraemia and endocarditis) [2]. The extensive use of antibiotics has resulted in the rise of multiresistant *E. faecalis* strains, making the treatment of infections difficult. The identification of traits that contribute to their pathogenicity is important in understanding the dual nature of this organism [24,46,47].

Despite scientific interest in this bacterium in recent years, the genetic toolbox and methodologies available for overexpressing genes of interest are more limited than for some other microorganisms [48]. Although heterologous gene expression in an *E. coli* background is the most common method of producing recombinant proteins from other bacteria, autologous gene expression is an alternative when heterologous expression has failed [49]. Availability of appropriate expression vectors for *E. faecalis* becomes essential to express cloned genes efficiently, to study regulatory proteins, or to carry genetic complementation studies [50]. A number of expression systems for *Enterococcus* are available. Probably the best known controllable expression system for Gram-positive bacteria is based on the autoregulatory properties of nisin biosynthesis in *L. lactis* [38,39,51,52]. The system can be used in a wide range of genera including *Lactococcus*, *Streptococcus*, *Enterococcus*, *Leuconostoc* and *Lactobacillus* [53]. However, when used in *Enterococcus*, the regulatory genes *nisR* and *nisK* need to be supplied *in trans*. Similarly, the enterocin regulatory system for controlling the expression of heterologous genes in *Enterococcus* requires the regulator- and kinase-encoding genes *entR* and *entK* be expressed *in trans* [32]. In order to tackle this disadvantage, the NICE system was optimised to be used in *Enterococcus* as a single-plasmid expression vector [34]. Other inducible expression vectors for *E. faecalis* are available: the rhamnose-inducible system [35] and the recently described one that is under control of the pheromone cCF10 [54]. The present work describes an alternative system for controlled gene expression in *E. faecalis* using a single-plasmid expression system.

The present results show putrescine accumulation by *E. faecalis* V583 to be strictly agmatine-dependent. They also show that *aguR* gene expression is not modified by an increase in agmatine concentration of the culture medium, whereas *aguBDAC* gene expression is significantly upregulated by agmatine concentrations of over 0.25 mM. The suitability of the *aguR*/P_*aguB*_ system (the regulatory part of the AGDI system of *E. faecalis* V583) as an agmatine-induced gene expression system was therefore explored. An expression vector combining the *aguR* activator gene and the *aguB* promoter, followed by convenient cloning sites for introducing the gene of interest, was constructed. An important feature of the developed pAGEnt system is the addition of a histidine tag, which makes the vector an option for protein purification purposes. By cloning the target gene into the *Nco*I-*Pst*I sites, the gene is inserted into the correct frame and fused to the His-tag. This His-tag was obtained from expression vectors previously shown to perform efficiently when used in protein overproduction, immunodetection and purification settings [40,55].

This expression system was assessed by expressing the reporter gene *gfp* (which codes for green fluorescent protein). Very strong fluorescence induction (40 arbitrary units compared to 0 in uninduced cells) was seen in the presence of 60 mM agmatine. It should be noted that agmatine concentrations of 60 mM are within the range associated with no toxic effect in *Enterococcus*. A potential benefit of having an expression system based on *aguR*/P_*aguB*_ is that gene expression can be finely controlled upon the addition of appropriate concentrations of agmatine. Agmatine is an intermediate in polyamine biosynthesis, and ubiquitous in living cells. However, it is normally present only in trace amounts - tissue concentrations are usually below 1 μM [56]. It should also be noted that this system requires the expression of no additional proteins supplied *in trans*.

The present results clearly show that this system can effectively control the expression of genes in response to non-toxic agmatine in *E. faecalis*. This could be a useful tool for the overexpression of proteins in this species, and expands the toolbox available to use with it. Moreover, this system may be active in other *Enterococcus* species and perhaps in other Gram-positive hosts (as the NICE system may be used), although this needs to be confirmed.

## Conclusions

The described agmatine-inducible system represents an attractive means for the overproduction and purification of recombinant proteins in *E. faecalis.* The present work describes the construction of an *E. faecalis aguR*/P_*aguB*_ controlled expression system and demonstrates its potential as a means of overproducing recombinant proteins. This system was assessed by expressing the reporter gene *gfp*, and very strong fluorescence was induced in the presence of 60 mM agmatine (40 arbitrary units compared to 0 in uninduced cells). The potential benefit of this system is that gene expression can be finely controlled by the addition of appropriate concentrations of agmatine. The addition of a histidine tag to the pAGEnt vector renders the system suitable for protein purification purposes.

## Methods

### Bacterial strains and growth conditions

*L. lactis* NZ9000 was grown at 30°C in M17 medium (Oxoid, Basingstoke, United Kingdom) supplemented with 30 mM glucose. *E. faecalis* V583 was grown at 37°C in M17 medium (Oxoid, Basingstoke, United Kingdom) supplemented with 30 mM glucose (GM17). When required, the indicated concentration of agmatine (Sigma-Aldrich, St. Louis, MO) was added to the medium. Chloramphenicol (5 μg ml^−1^) was added as required.

### Analytical chromatography methods

Cultures were centrifuged at 8000 *g* for 10 min and the resulting supernatants filtered through a 0.2 μm Supor membrane (Pall, NY). Putrescine and agmatine concentrations were analysed by ultra-performance liquid chromatography (UPLC™) using a Waters H-Class ACQUITY UPLC™ apparatus controlled by Empower 2.0 software and employing a UV-detection method based on derivatization with diethylethoxymethylene malonate (Sigma-Aldrich), as previously described [57].

### DNA manipulation procedures

The procedures used for DNA manipulation and recombination were essentially those described by Sambrook *et al.* [58]. Table 1 lists the primer sequences used. Genetic constructs for *Enterococcus* were achieved using *L. lactis* NZ9000 as an intermediate host. Plasmid and total DNA of *L. lactis* and *Enterococcus* were isolated and transformed as previously described [59]. All plasmid constructs were verified by nucleotide sequencing at Macrogen Inc. (Seoul, Republic of Korea). All enzymes for DNA technology were used according to the manufacturer’s specifications.

### RNA extraction

Total RNA was extracted at 6 h of incubation using the TRI Reagent (Sigma) as previously described [21]. *E. faecalis* was grown in M17 or in M17 supplemented with 30 mM glucose and 0, 0.05, 0.1, 0.25, 0.5, 1, 5, 10 or 20 mM agmatine. The cells were then harvested by centrifugation and disrupted using glass beads (diameter up to 50 μm) in a Fast-Prep FP120 apparatus (Thermo Savant-BIO101/Q-Biogen) at 4°C for 6 × 30 s (power setting 6). The resulting samples were treated as recommended by the manufacturer. Purified RNAs were resuspended in RNAse-free water. After extraction, RNA samples were treated with DNase (Fermentas, Vilnius, Lithuania), as described by the manufacturer, to eliminate any genomic contamination. Total RNA concentrations were determined by UV spectrophotometry by measuring absorbance at 260 nm in a BioPhotometer (Eppendorf, Germany).

### Gene expression quantification by RT-qPCR

Gene expression analysis was performed by reverse transcription-quantitative PCR (RT-qPCR) in a 7500 Fast Real-Time PCR System (Applied Biosystems, Carlsbad, CA) using SYBR® Green PCR Master Mix (Applied Biosystems). After 2-fold dilution of the cDNA, 5 μl were added to 20 μl of PCR mixture (12.5 μl of SYBR Green Supermix, 1 μL of each primer at 7 μM, and 5.5 μl of RNAse-free water). Amplifications were performed with specific primers (Table 1) designed with Primer Express software (Applied Biosystems); primers specific for elongation factor thermo-unstable (*tufA*) and recombinase A (*recA*) genes were used as references. The cycling settings were those default-established by Applied Biosystems. For each condition, RT-qPCR analysis was performed on RNA purified from three independently grown cultures.

### Green fluorescence measurements

For whole-cell fluorescence measurements, equal amounts of cells were harvested, washed and subsequently resuspended in 50 mM KPi, pH 7.2, as previously described [40]. GFP emission was measured in a volume of 200 μl of cells, using a Cary Eclipse fluorescence spectrophotometer (Varian Inc., Palo Alto, CA), at an excitation wavelength of 485 nm and an emission wavelength 530 nm. For direct comparison, all the GFP fluorescence data were normalized to the same *A*_600_. Background fluorescence levels were assessed by measuring non-fluorescent control cells, and these values subtracted.

### Statistical analysis

Student *t* tests were used to evaluate the consistency of the data.

## Abbreviations

AGDI: Agmatine deiminase
GFP: Green fluorescence protein
VRE: Vancomycin resistant *Enterococcus*
RT-qPCR: Reverse transcription quantitative PCR
CFU: Colony forming units
